# Virtual Microscopy Tagging and Its Benefits for Students, Faculty, and Interprofessional Programs Alike

**DOI:** 10.7759/cureus.27860

**Published:** 2022-08-10

**Authors:** Katsiaryna Khatskevich, Yoon Seon Oh, Daniel Ruiz, Britton McGlawn-McGrane, Gabriana Freire, Langfeier Liu, Nicholas Lewis, Rahul Mhaskar

**Affiliations:** 1 Pathology and Laboratory Medicine, Medical University of South Carolina, Charleston, USA; 2 Morsani College of Medicine, University of South Florida Morsani College of Medicine, Tampa, USA; 3 Internal Medicine, Medical University of South Carolina, Charleston, USA; 4 Internal Medicine, University of South Florida Morsani College of Medicine, Tampa, USA

**Keywords:** organization, histology, curriculum, updated technology, virtual microscopy

## Abstract

Background

Over the years, due to technological innovations in medical education, virtual microscopy has become a popular tool used to teach histology. The Virtual Microscopy Project is a faculty and student collaborative project at the University of South Florida (USF) Health to transfer and update online histology slides from an outdated viewer to a completely new viewer.

Methodology

The project goal is to better facilitate the educational experience for students and faculty through the implementation of updated technology and features.

Results

At USF Health, multiple programs use the online histology slide viewer to teach normal histology. The previous website’s organization and lack of additional features severely hindered opportunities for personalized education; users could not write any notes, circle or point to important features, or easily search for specific organs or tissue. An updated website user interface and additional instructional features will improve the students’ accessibility and overall quality of their learning. The updated viewer will be more integrated into the USF Health Morsani College of Medicine (MCOM) curriculum, providing faculty with organized and readily available material. USF MCOM has faculty integration directors to create a balanced curriculum that can be reviewed by faculty for consistency and accuracy. Adding this same organizational structure to the online microscopy viewer will assist directors in forming and modifying the curriculum and may also provide them with additional resources for their education delivery.

Conclusion

The Virtual Microscopy Project hopes to produce an accessible, user-friendly online microscopy viewer that is beneficial to students for learning, to faculty for teaching/curriculum planning, and to medical education as a whole.

## Introduction

Currently, there are various studies reporting the significance of virtual microscopy websites in increasing student accessibility to histology slides; however, there are no studies on virtual microscopy websites that also incorporate features useful for faculty in developing and teaching curricula [1, 2]. The Virtual Microscopy Project is a faculty and student collaborative project at the University of South Florida (USF) Health with the purpose of transferring and updating online histology slides from an outdated viewing interface to a new and improved version designed from the ground up. The intent of this project is to ultimately better facilitate both the teaching and learning experience for faculty and students through the implementation of an updated user interface by not only increasing accessibility to histology slides but also by integrating organizational tags based on the school curriculum. USF Health incorporates multiple programs (College of Medicine, Physician Assistant Program, Anatomy Masters Program), all of which use the same online histology slide viewer for normal histological education. An updated website user interface with additional instructional features will not only increase the ease of accessibility to and within the site but also improve the overall quality and efficiency of their learning [3-10]. Furthermore, the updated virtual microscopy viewer can smoothly integrate into the USF Health Morsani College of Medicine (MCOM) curriculum, providing faculty with organized and readily available material at their disposal [11]. USF MCOM has select faculty integration directors to create and sustain a balanced curriculum over the four years, which is reviewed for consistency and efficacy. Incorporating this same organizational structure into the online microscopy viewer will assist faculty integration directors in forming and modifying the curriculum more quickly and comprehensively, as well as prove beneficial to teaching faculty by providing additional resources for their education delivery [12-14]. The goal of the virtual microscopy project is to create a convenient and informative online microscopy viewer that is beneficial to students and faculty by allowing personalized education and increasing ease of accessibility.

## Materials and methods

The Virtual Microscopy Project consists of multiple teams. The software/IT team focused on the technical aspects of creating the new website. The USF Health faculty team is responsible for bringing the teaching perspective to delineate which histology slides are necessary for the website. The USF Health MCOM student team worked toward organizing and tagging each slide while offering the perspective of a student using the website. The student team consisted of two third-year medical students and three second-year medical students. This was a qualitative case study of a single institute.

**Figure 1:**
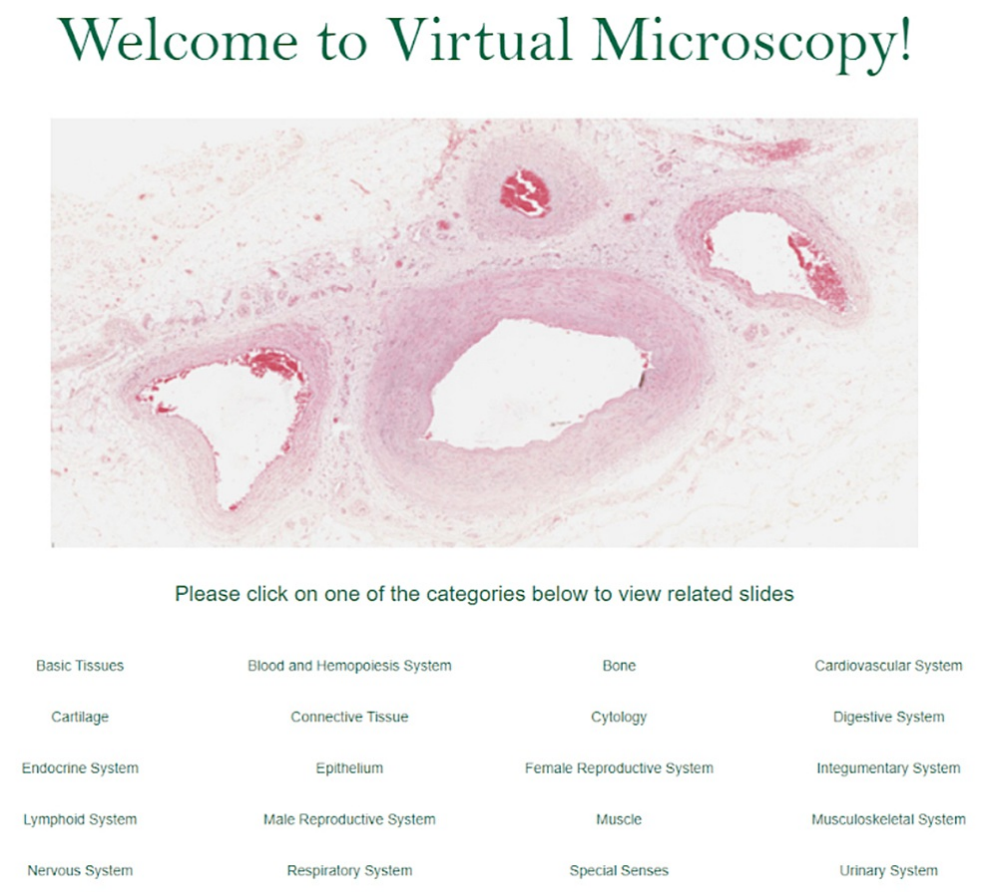
New website home page, organized by organ system. The histology slide was uploaded by Karl Muffly 2020. Photographed by the author, Katsiaryna Khatskevich, in 2020.

The student team collaborated with USF Health faculty to brainstorm features students would like to see in the completed website, while the software team began setting up the foundation for the new website. Once all of the histology slides were selected for the new website, they were organized in a categorical system so that it could encourage ease of access and intuitive understanding for all students within USF Health, regardless of the program (Figure 1). The slides were first organized by organ system and then by normal histology versus abnormal histology within each organ system. While all initial slides were normal histology, this model of organization was chosen so that space could be left in the organizational schemata for abnormal histology slides to be added in the future. This also allows for students to compare and contrast normal histological presentations to those that correspond to different pathology. After the organizational system was put in place, we tagged each slide with a set of keywords. The purpose of tagging in addition to the categorical organization was so anyone could visit the website and use the search bar to find the appropriate slide(s) based on general keywords (e.g., stratified squamous, heart valve, skeletal muscle), such that students could quickly find relevant slides. Initially, we wanted to tag using simple keywords only. We later decided to include tags that would be especially beneficial to faculty. We discussed this with several MCOM faculty integration directors, who are charged with creating and maintaining the balance and consistency of course content across all four years of the MCOM curriculum. Specifically, each integration director is responsible for mapping the existing curriculum and finding the gaps and redundancies within the curriculum that correspond to their designated content area. Then, with the consultation of other involved faculty, they are to revise the content to achieve the educational goals for USF MCOM. Each course of study (i.e., anatomy, pathology, pharmacology, etc.) has unique national curriculum objectives that programs can use to keep track of the expected content covered within undergraduate medicine programs. Some content areas, such as histology, do not have national objectives. Instead, we used the histology objectives designed by MCOM histology integration director, Dr. Marzenna Wiranowska. These curriculum objectives, used for curriculum mapping by the USF MCOM faculty, were used to tag the slides. Hence, users of the virtual microscopy website can now search for a specific concept that is a component of the four years of the MCOM MD program curriculum (e.g., liver cirrhosis) and see which slides relate to that particular concept. This tagging allows students to review the course content along with the related slides from the virtual microscopy repository. Moreover, the tagged slides serve as a useful resource for faculty to integrate the slides into their content and lectures. Overall, the tagging paves the way for seamless integration of content across courses throughout all four years of the MD program curriculum.

Curriculum directors assign objectives to all modes of educational content delivery: PowerPoint lectures, in-person laboratories, group projects, etc. Using standardized objectives, a curriculum director can see the frequency and manner in which a particular subject is taught, which allows them to quantify if a subject is being taught too often, not often enough, or is not balanced across all schooling years. We collaborated with the histology, physiology, and anatomy integration directors specifically and received the objective tags they used or developed. The objectives were incorporated as tags for the online histology slides, as applicable to each one. The ability to track the objectives via tagging allows integration directors to utilize the virtual microscopy viewer to readily deliver the content and incorporate it into the curriculum.

In addition to the integration directors, teaching faculty also use the same objectives in creating their PowerPoints and lectures. Staying consistent with the same tagging system allows teaching faculty to easily find histology slides they can use to support the objective they are teaching in lectures. This has the added bonus of also being useful for students; all lecture PowerPoints at USF MCOM have an “objectives” slide specific to the lecture. Students will be able to search a lecture’s objectives within the microscopy viewer to find the same slides used in the PowerPoint in addition to other relevant slides that may help reinforce the same objective topic. Incorporating the curriculum objectives into tags would benefit students, teaching faculty, and the design of an integrative curriculum.

## Results

**Figure 2:**
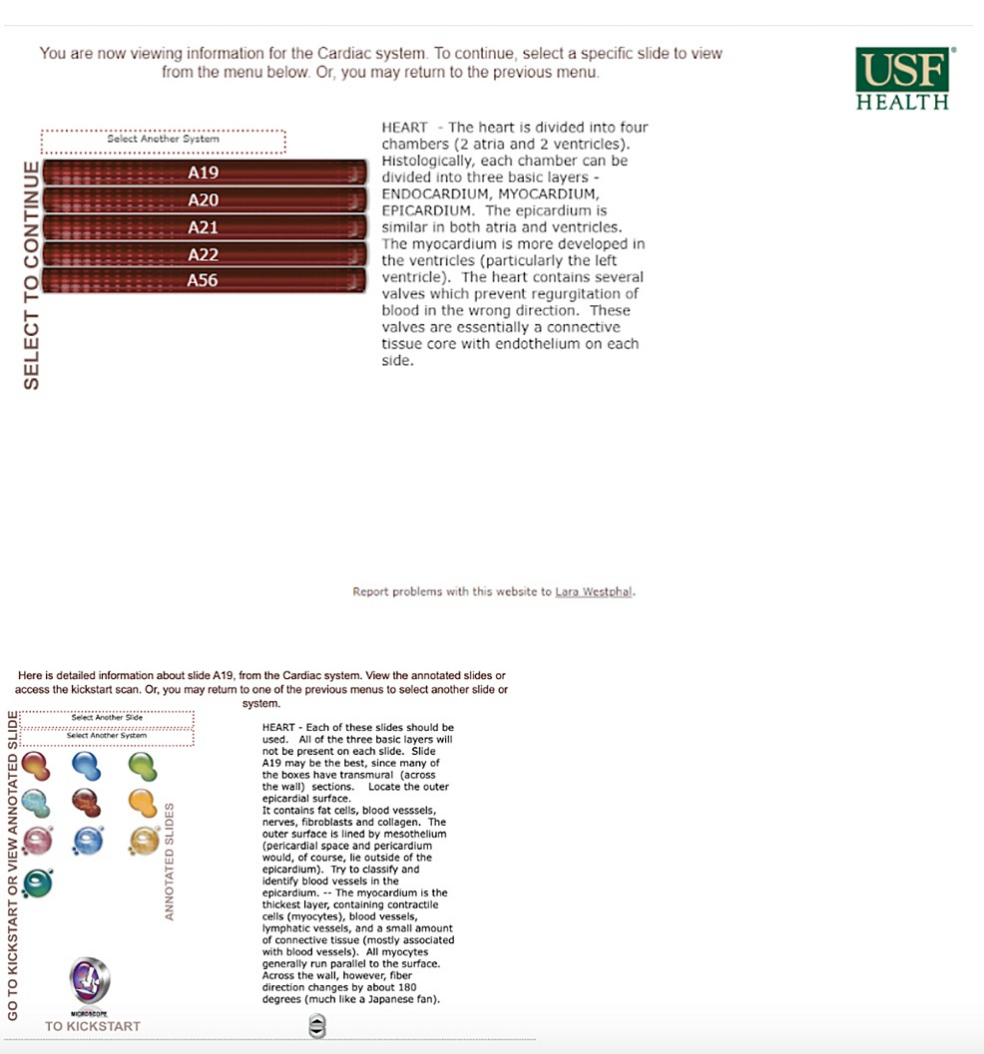
Old website Cardiac Histology designed by Karl Muffly. Photographed by the author, Katsiaryna Khatskevich, in 2020.

**Figure 3:**
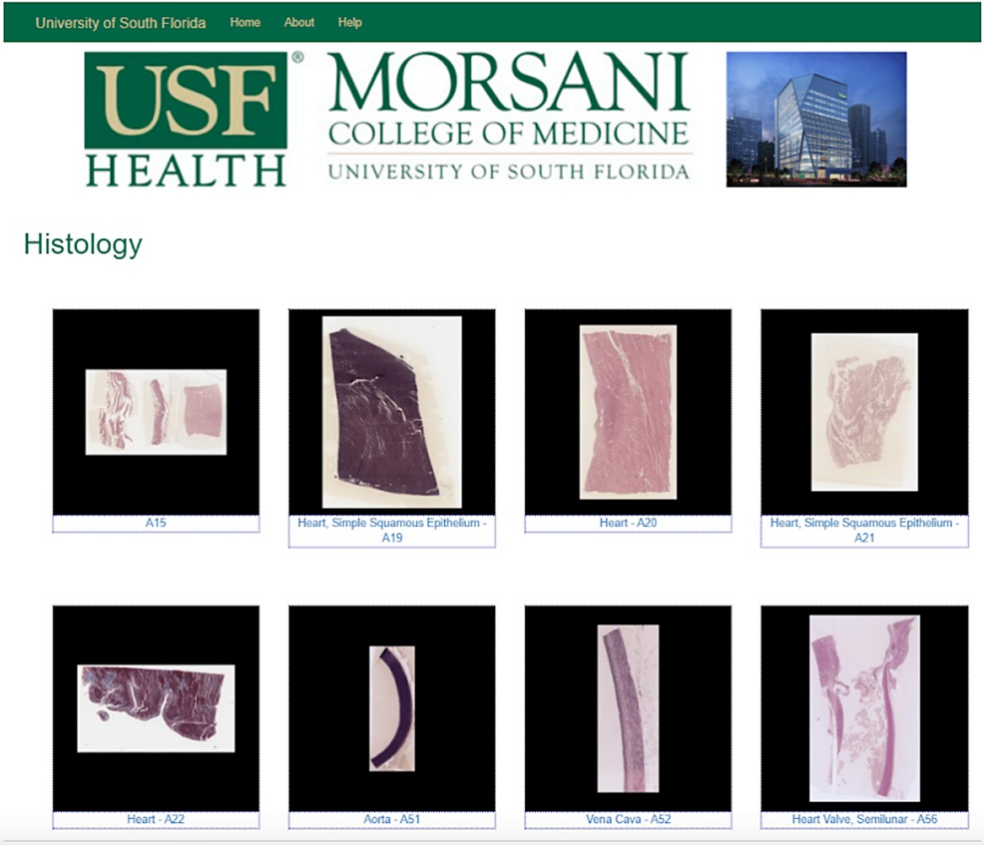
New website Cardiac Histology. The histology slides were uploaded by Karl Muffly 2020. Photographed by the author, Katsiaryna Khatskevich, in 2020.

The end product is a microscopy viewer that offers new learning tools that students and faculty can utilize throughout the curriculum. Compared to the previous website, which was organized solely by organ system and then all slides that belonged to it (Figure 2), the new website has a new organization system decided on by a committee of students and faculty. It is organized by organ system but also further sorted by organ and whether the slide had normal or pathologic tissue. It displays slides named by the organ/tissue of interest, instead of the filing letter/number the previous website used (Figure 3). The incorporation of the tagging system, as well as the addition of the new slide titles, allows viewers to locate slides in a variety of ways. They can now search for a specific slide title, a tag of a structure featured in multiple slides, or a curriculum objective.

**Figure 4:**
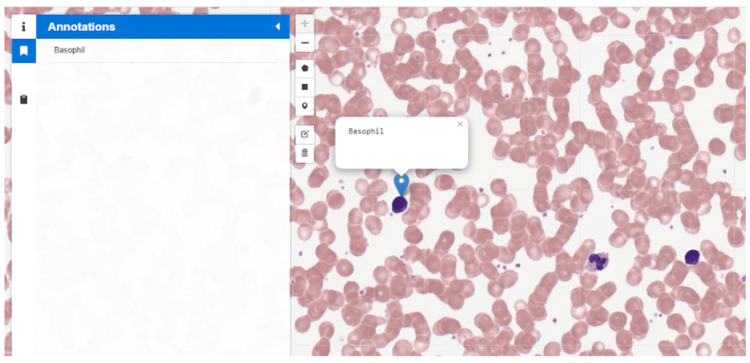
The new website allows a user to highlight areas or drop a marker and label the point of interest. The histology slide was uploaded by Karl Muffly 2020. Photographed by the author, Katsiaryna Khatskevich, in 2020.

In addition to the new navigation schemata, there are new features available for students and faculty. On the old website, clicking on a slide only permitted users to scroll across the slide and zoom in and out with considerable lag time. The new website, in addition to smooth and responsive scrolling/zooming, also allows users to highlight relevant areas, leave custom markers for areas of interest, and create notes for a highlighted area or the entire slide as a whole (Figure 4). Students and faculty can mark specific areas, leave notes on a slide, and subsequently save that annotated copy. Students can use this copy to study from personalized notes tailored to their curriculum content. Faculty can use an annotated copy to teach from, highlighting points of interest on the slide that are pertinent to the specific course or lecture they are teaching. Faculty can also upload annotated copies to the viewer so that their students can use them to study. The new microscopy viewer offers many more tools than the previous viewer did which, in turn, provides more opportunities for interactive use from students. Integration directors can use the tagging system to search for curriculum objectives to find ones that are repeated or lacking within the curriculum. Teaching faculty can also search by the tags to add screenshots of relevant slides to their presentations or simply offer the viewer as a general resource to students.

## Discussion

The new software has enabled the histological component of the pre-medical education at USF Health to become streamlined and has encouraged significantly more use from students and faculty when compared to the prior iteration. For lectures focused on histology, students are briefly given a foundational understanding of the variations between normal histology and abnormal histology before exploring the viewer as a group in an “engaged learning” session. Studies on information and communication technologies (ICT) have shown that the incorporation of ICT can increase student participation and collaborative learning [15]. These sessions, and in turn this software, have enabled students to expand their understanding of histological concepts. As an example, students would previously learn to identify a histological element such as a neutrophil based on an example given in a lecture slide. While this may suffice for simpler elements, others can have significant variation from slide to slide, and thus a student’s ability to identify said element in different contexts will be insufficient. This new software instead enables a student to view several different variations of the same element to gain a more holistic understanding of how to identify it under different conditions at their convenience [16]. This holistic approach to histological education is now both incorporated into the curriculum through the aforementioned “engaged learning” sessions, as is additionally highly encouraged for students to engage in through self-directed learning [17,18]. This project offers a localized hub and continuity for students among professors, lecture PowerPoints, and histology slides found on this website. The new website has benefitted curriculum integration directors in curriculum mapping and teaching faculty in functioning as an additional teaching tool.

This project consisted of using only the histology slides found on the old website, and, while the content itself was not changed, the process of transitioning them to a new website platform had additional benefits. In addition to having the opportunity to rearrange the slides, some missing tissues were also identified (i.e., the old website had no slides of parathyroid tissue) which are now being worked on to locate and upload. The updated virtual microscopy viewer was also found to be compatible with other educational tools. The images of the slides can be uploaded onto Poll Everywhere, an interactive website used to quiz students and see their responses in real-time. When used in conjunction with Poll Everywhere, the updated website may increase in class participation and the students’ understanding and retention of histological concepts can be checked and monitored live. The use of an up-to-date website platform may also increase the speed of the website and loading of the viewer, something that was previously an issue when over 100 students attempted to load the same webpage at once. In this way, the project can help streamline classroom learning from the technological aspect as well. The future goals of the study are to obtain data on student use of the virtual microscopy website, student opinion on the website, and ease of access and utilization of the website.

The future of this project aims to add new slides to the viewer to compare and contrast normal and abnormal histology and hopefully develop a diverse enough database to be of benefit for training pathology residents. The website currently consists of only normal histology, and, while this is useful for first-year medical students (whose curriculum focuses on normal anatomy and physiology), it is less useful for other medical students, master’s students, and residents. The flexibility of the new layout will allow for the accommodation of new abnormal histology slides in the future. The hope is to continue to expand this website and be able to implement new slides into the already existing organization scheme to provide students with as many different examples as possible.

## Conclusions

The virtual microscopy viewer utilized by USF Health to teach and expose students to histology was updated with categorical organization, tagging, and the ability to search for and interact with specific slides. In addition, with curriculum adjectives as tags, the website was formatted to be of additional assistance to faculty and curriculum directors alike by linking classroom learning to the website as well as balancing the curriculum. The educational experience of using normal histology slides can be further enhanced in the future with the addition of abnormal histology slides, which would allow users to compare and contrast normal and abnormal histology. The addition of this feature would transform the virtual microscopy viewer into a resource where all histology slides used in classes can be found, which, in turn, would streamline the teaching and learning process.
